# Development and On-Field Deployment of a Mobile-Based Application ‘MoSQuIT’ for Malaria Surveillance in International Border Districts of Northeast India—Challenges and Opportunities

**DOI:** 10.3390/ijerph19052561

**Published:** 2022-02-23

**Authors:** Saurav Jyoti Patgiri, Gunenja Gobinda Gohain, Santanu Kumar Goswami, Dibya Ranjan Bhattacharyya, Sudhanshu Hari Das Debnath, Lakshmi Panat, Ganesh Karajkhede, Pradyumna K. Mohapatra, Devojit Kumar Sarma, Ipsita Pal Bhowmick, Kongkona Gogoi, Sujit Biswas, Jayanta Debnath, Sukanta Acharjee, Susmita Senapati, Rahul Neog, Prabal Nath, Keisham Meitei, Subrata Baidya, Dinesh Debbarma, Ajit Sarma, Rahim A. Ahmed, Hemkanta Boro, Rubal Chandra Das, Jagadish Mahanta, Satya Ranjan Debbarma, Harpreet Kaur

**Affiliations:** 1 ICMR-Regional Medical Research Centre, North East Region, Dibrugarh 786010, India; gunenjagohain@gmail.com (G.G.G.); skgoswami.rmrcne@gov.in (S.K.G.); drbhattacharyya@yahoo.com (D.R.B.); pkmdibr@gmail.com (P.K.M.); ipsita.icmr@gmail.com (I.P.B.); koko1.gogoi@gmail.com (K.G.); biswassujit83@yahoo.in (S.B.); debnathjayantabsc@gmail.com (J.D.); sukantaacharjee75@gmail.com (S.A.); senapatitiku999@gmail.com (S.S.); rahul.neog@gmail.com (R.N.); prabal.nath10@gmail.com (P.N.); keishammeitei@gmail.com (K.M.); jmahanta@gmail.com (J.M.); 2 Centre for Development of Advanced Computing (C-DAC), Pune 411007, India; sudhanshud@cdac.in (S.H.D.D.); lakshmip@cdac.in (L.P.); ganeshk@cdac.in (G.K.); 3 ICMR-National Institute for Research in Environmental Health, Bhopal 462030, India; dkbiotek@gmail.com; 4 Department of Community Medicine, Agartala Government Medical College, Agartala 799006, India; drsubratabaidya@gmail.com; 5 National Vector Borne Disease Control Programme, Dhalai 799289, India; barmandinesh87@yahoo.com (D.D.); nvbdcpdhalai@gmail.com (A.S.); 6 National Vector Borne Disease Control Programme, Udalguri 784509, India; rahim.ahmed@gmail.com; 7 Department of Health & Family Welfare, Tamulpur, Baksa 781367, India; mts.tamulpur.baksa@gmail.com; 8 Changlang District Hospital, Changlang 792120, India; dvbdcpchanglang@gmail.com; 9 Indian Council of Medical Research (ICMR), Agartala 799006, India; satyaranjandebbarma@yahoo.com; 10 Indian Council of Medical Research (ICMR), New Delhi 110029, India; kaurh.hq@icmr.gov.in

**Keywords:** malaria, MoSQuIT, surveillance, NVBDCP, *Plasmodium falciparum*, *Plasmodium vivax*, Northeast India

## Abstract

The conventional paper-based system for malaria surveillance is time-consuming, difficult to track and resource-intensive. Few digital platforms are in use but wide-scale deployment and acceptability remain to be seen. To address this issue, we created a malaria surveillance mobile app that offers real-time data to stakeholders and establishes a centralised data repository. The MoSQuIT app was designed to collect data from the field and was integrated with a web-based platform for data integration and analysis. The MoSQuIT app was deployed on mobile phones of accredited social health activists (ASHA) working in international border villages in the northeast (NE) Indian states of Assam, Tripura and Arunachal Pradesh for 20 months in a phased manner. This paper shares the challenges and opportunities associated with the use of MoSQuIT for malaria surveillance. MoSQuIT employs the same data entry formats as the NVBDCP’s malaria surveillance programme. Using this app, a total of 8221 fever cases were recorded, which included 1192 (14.5%) cases of *P. falciparum* malaria, 280 (3.4%) cases of *P. vivax* malaria and 52 (0.6%) mixed infection cases. Depending on network availability, GPS coordinates of the fever cases were acquired by the app. The present study demonstrated that mobile-phone-based malaria surveillance facilitates the quick transmission of data from the field to decision makers. Geospatial tagging of cases helped with easy visualisation of the case distribution for the identification of malaria-prone areas and potential outbreaks, especially in hilly and remote regions of Northeast India. However, to achieve the full operational potential of the system, operational challenges have to be overcome.

## 1. Introduction

The global burden of malaria stood at 241 million cases in 2020, which was an increase of 6.2% over the previous year. Malaria-related mortality also increased by around 12% over the same period [1]. Service disruption during the COVID-19 pandemic was one of the main factors contributing to this increase in malaria cases and deaths; earlier reports had shown a declining trend in most malaria-prone areas. The WHO South-East Asia Region contributed approximately 2% to the global burden of malaria in 2020. Interestingly, India accounted for roughly 83% of the cases in the WHO South-East Asia Region, which is a matter of concern. In times like these, when communication and services are disrupted, digital data management platforms and app-based data collection tools are vital to ensure timely data collection and transmission of malaria surveillance data.

Northeast India, which includes the states of Assam, Meghalaya, Arunachal Pradesh, Manipur, Nagaland, Mizoram, Tripura and Sikkim, contributes over 7% of India’s total malaria burden although accounting for only 3.76% of the country’s population [2,3]. This region is strategically located, with Southeast Asian countries, such as China and Bhutan in the north, Nepal in the west, Myanmar in the east and Bangladesh in the southwest sharing over 99 percent of its total geographical boundary [4]. The heterogeneity of the terrain, as well as the diversity of the people residing in this region, makes malaria management a difficult task. The malaria-endemic areas along NE India’s international borders are hilly and remote. As per the guidelines of the National Vector Borne Disease Control Program (NVBDCP), there are different paper-based forms for data collection, reporting and calculating malaria indicators. Active surveillance necessitates health workers travelling significant distances to field sites to collect data on paper-based M1 forms. The same paper-based form must be returned to the health centres to be submitted. Paper-based data is manually moved from the health centre to the district level at several stages. At different stages, manual data collection is carried out. Reports and epidemiological indicators are calculated based on the manually collected available data. This paper-based approach is extremely difficult to track and manage. This could cause a delay in taking appropriate actions. We attempted to convert this paper-based method to MoSQuIT, which is a mobile phone and web-based malaria surveillance system, to overcome this problem. MoSQuIT provides real-time data to the stakeholders and produces a centralised data repository that is readily accessible and interpretable, allowing decision makers and policymakers to make decisions based on real-time clinical data.

The Mobile-based Surveillance Quest using IT (MoSQuIT) is Android-based mobile software for real-time malaria surveillance that was developed by the ICMR—Regional Medical Research Centre, North East Region, Dibrugarh, Assam, in partnership with the Centre for Development of Advanced Computing (C-DAC), Pune. The platform was originally developed in 2011 and evaluated in 50 villages in Assam’s Dibrugarh district between 2012 and 2013. [5]. The pilot project demonstrated promising results, prompting continued development and wide-scale deployment of the application across Northeast India’s international border regions in 2017. In recent years, mobile-phone-based surveillance has been used for health-related activities around the world [6]. In India, the use of mobile platforms for collecting malaria surveillance data is a relatively new trend, with only a few districts and state health agencies collaborating with public health experts to implement the platform. Since 2015, the Malaria Control System (MCS) has been used in Mangaluru, Karnataka, for malaria data collection, mapping and monitoring activities, and has demonstrated sustained reduction in malaria incidence over five years. This system collects surveillance data through an Android tablet with in-built geographical information system (GIS) tagging and transmits the data, which can be accessed through a web-based system [7]. Similarly, a mobile application tool known as SOCH (Solution for Community Health Workers) was implemented in 2017 for real-time malaria surveillance and reporting in the Mandla district of Madhya Pradesh [8]. Even in Northeast India, the FeverTracker app has recently been deployed for active malaria surveillance in 19 tribal villages in Tripura’s Dhalai district [9].

The present study describes the design, development and on-field deployment of the MoSQuIT (Mobile-based Surveillance Quest using IT) application in malaria-endemic districts of three international border regions of Northeast India. The deployment results, along with the sequential scale-ups in the platform and the challenges faced during the process, are also discussed.

## 2. Materials and Methods

### 2.1. Design and Development of the MoSQuIT app

**Figure 1:**
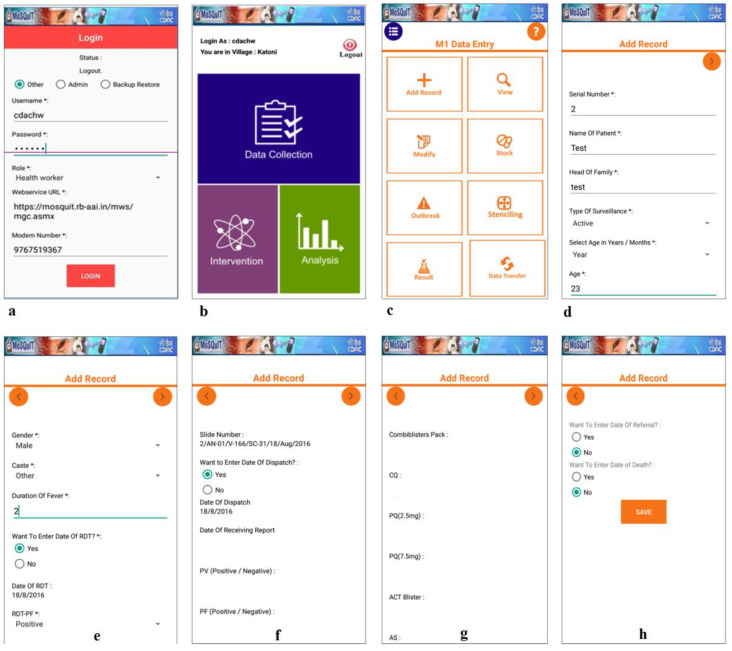
(**a**–**h**) Interface of the mobile-based MoSQuIT app (* refers to mandatory fields).

The MoSQuIT application and web portal were designed to facilitate accurate data entry and analysis of malaria surveillance data. The mobile application was developed on J2ME and upgraded to Android 5.1 using Android Studio. For ease of operation and to conform to the currently used data entry formats used for malaria surveillance under NVBDCP, the mobile interface was kept similar to conventional paper-based formats used by the healthcare workers on a routine basis. The M1, M2, M3 and M4 forms used for routine surveillance were adapted to the mobile interface using clean and easily comprehensible pages with drop-down and selectable fields and scrollable windows with a minimum requirement for manual typing (Figure 1a–h). Although the current app interface was developed for *P. falciparum* and *P. vivax*, other malarial parasites can be included in the future as and when encountered. It is to be noted that most of the rapid diagnostic kits used under NVBDCP can detect only these two *Plasmodium* species. Similarly, to keep the app interface simple, clinical signs and symptoms were kept at a minimum, with the possibility of inclusion at a later stage. The major components of the mobile app were data entry modules for malaria surveillance, vector control and stenciling, along with geotagging of case records. In addition, the mobile app also included integrated ICT-based modules for continuous medical education, community awareness and participation.

### 2.2. Development of the Web-Based Portal

The MoSQuIT website and web service were developed in ASP.Net and VB.Net with the .Net Framework 3.2 using Visual Studio 2008. This system was deployed on Windows Server 2012 and IIS version 8. The IVRS component was developed in C#. It uses Dialogic^®^ JCT Media Boards and a PRI line from any service provider for sending IVRS calls and receiving a response. The modem component was developed in VB.Net. Validations for the mobile and web interfaces were successfully achieved as per the software requirement specifications. The testing of mobile and web applications strictly followed test cases and generated test instances with a bug report. Both mobile and web applications were subjected to third-party security audits using tools like Subgraph Vega and Acunetix. After successful remediation of the mentioned threats in the audit report, a security certificate was issued for validity for one year.

**Figure 2:**
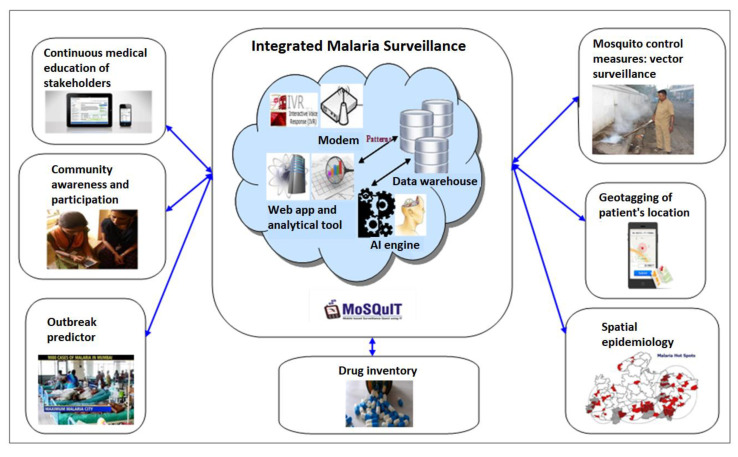
Components of the MoSQuIT application and web-based platform.

**Figure 3:**
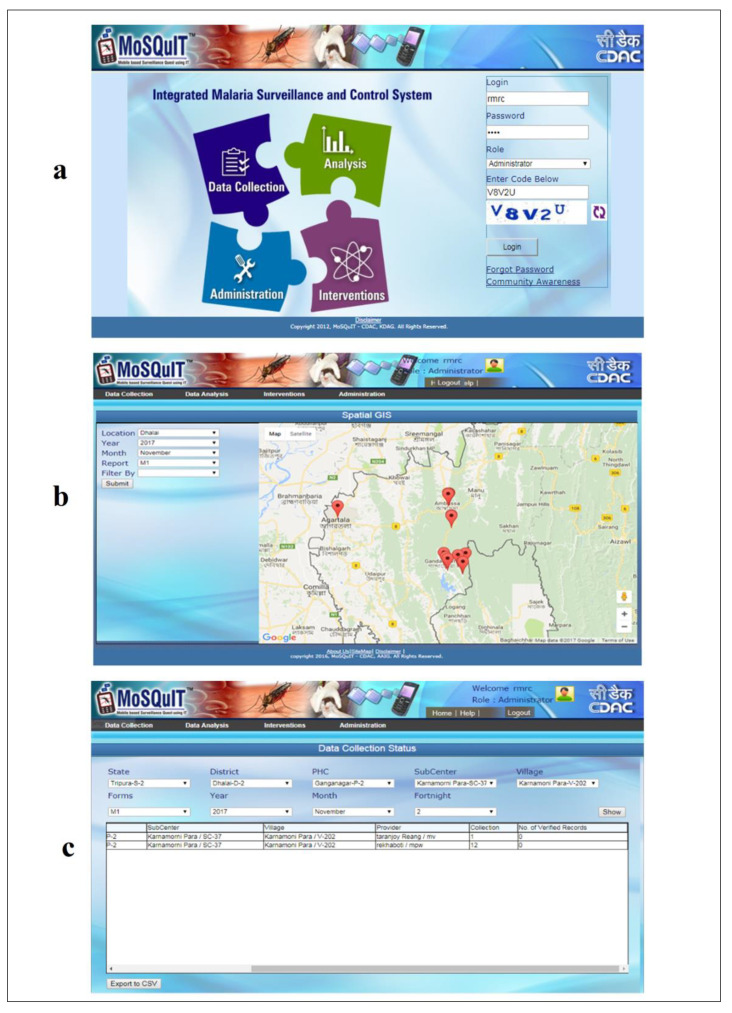
Snapshots of the web-based portal linked to the MoSQuIT application. (**a**) Login page of the web-based portal (**b**) Spatial GIS visualization panel (**c**) Village level data collection status.

Briefly, the web-based system components included those in the mobile app along with a few other additions such as Outbreak predictor, spatial epidemiology module and the analytics module, as shown in Figure 2 below. Snapshots of the web-based app are shown in Figure 3a–c below.

### 2.3. Framework for Data Collection, Collation and Analysis

**Figure 4:**
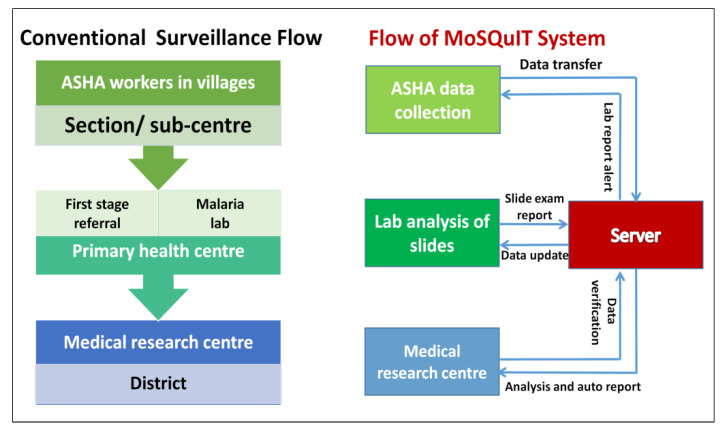
Data flow in the conventional paper-based surveillance system vs. MoSQuIT (figure adapted from Reference 5. Licence: CC BY-NCSA 3.0 IGO).

**Figure 5:**
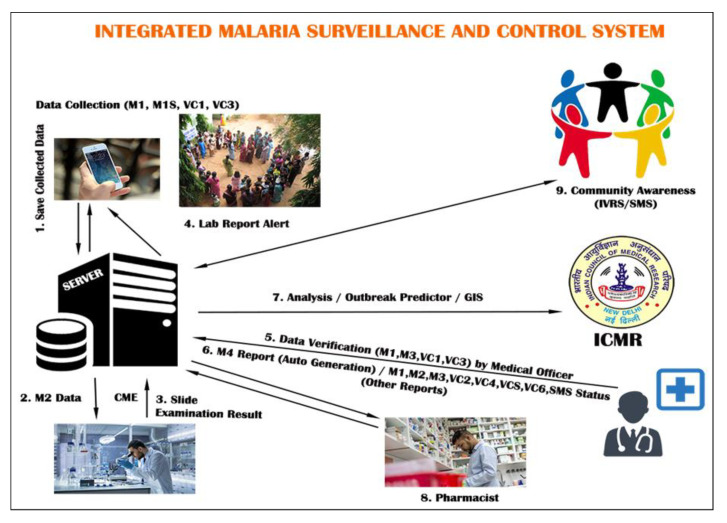
Overview of the MoSQuIT framework.

In the conventional paper-based system, data flow occurs in the following sequence: from health worker → sub-centre → primary health centre → district. In MoSQuIT, data flow occurs directly from the accredited social health activists (ASHA) and lab technicians to the server system at ICMR-RMRC, Dibrugarh, where the data is stored and analysis can be done (Figure 4) [5]. Data is collated on the server, which can be validated by medical officers. The same data is accessible by the stakeholders comprising of medical officers, laboratory technicians and health authorities for swift action. These stakeholders can log in to the web-based portal and view the records daily. Decision makers get automated reports and epidemiological indices on fortnightly, monthly, quarterly and annual bases. The real-time data can be used for generating epidemiological reports, case management and timely intervention in outbreak situations. MoSQuIT facilitates the following types of automated data analysis/reports: (i) statistical analysis based on malaria indicators (annual blood examination rate, annual parasite incidence, annual falciparum incidence, etc.); (ii) trend analysis based on person, place and time; (iii) efficiency analysis to measure the time lag; (iv) outbreak analysis; and (v) predictive analysis. A brief overview of the framework is provided in Figure 5. To ensure completeness of the data, the mobile app interface provides some compulsory fields, which have to be filled mandatorily before the case record can be saved and transmitted.

### 2.4. On-Field Deployment

This platform was deployed in a phased manner in selected villages belonging to four districts in three states of Northeast India. The study sites were located within 50 kilometres of the international border. These villages were located along the Indo–Bhutan border in the Baksa and Udalguri districts of Assam, Indo–Bangladesh border in Dhalai district of Tripura and Indo–Myanmar border in Changlang district of Arunachal Pradesh.

#### 2.4.1. Deployment in Dhalai District of Tripura along the Indo–Bangladesh Border

In the first phase, the Dhalai district of Tripura was the deployment area. An onsite survey was carried out in May 2017 and the following three areas along the international borders were selected after discussion with the local district authorities at Ambasa, the district headquarter of Dhalai district: (i) Ganganagar PHC area, (ii) Gandachara SDH and Dalapati PHC. The availability of mobile networks was also assessed in the region, which was found to be limited, with some areas having very poor to no mobile network coverage. The study area along the international border was remote, with poor road connectivity and access was difficult. Following the procurement of Android mobile handsets and the on-site survey in Tripura, the on-field deployment was started in September 2017. A two-day hands-on training was held at Ambasa in the Dhalai district of Tripura, involving around 28 health workers. On completion of the two-day training, mobile handsets with pre-activated SIM cards were provided to the health workers for operation in the field. The areas under coverage are detailed in Supplementary Table S1. Briefly, under the Ganganagar PHC area, a total number of 18 villages (paras) with a combined population of 5714 under two sub-centres, viz. Malda Kumar Para and Karnamoni Para, were included in the study. In the Gandachara SDH area, 20 villages with a combined population of 7720 under three sub-centres, viz. Bhagirathpara, Wanasapara and Ratan nagar SC, were included. In the Dalapati PHC area, the Dalapati SC, with a total population of 3000 in ten villages, was included.

#### 2.4.2. Deployment in Assam along the Indo–Bhutan Border

In the second phase, the study areas selected were the Udalguri and Baksa districts of Assam along the Indo–Bhutan border. The ICMR-RMRC team visited the Udalguri and Baksa districts of Assam on 16–21 January 2018. Based on the epidemiological reports obtained from the local health authorities and the malaria situation that was prevalent in the area, Udalguri BPHC and Orang PHC areas in Udalguri district and Tamulpur PHC area in Baksa district were selected for the study. The onsite training in the two districts of Assam was conducted jointly by the ICMR-RMRC N.E.R., Dibrugarh and C-DAC teams. The first training was organised at Udalguri from 20 to 21 March 2018. Altogether, 15 health workers, including multi-purpose health workers (MPW), ASHA, lab technicians and social workers, were trained and mobile phones were issued. The second training was held in Baksa from 22 to 23 March 2018. In Baksa, 15 health workers were trained and mobile phones were distributed at the end of the training. The deployment areas in these two districts are shown in Supplementary Tables S2 and S3. Briefly, in the Baksa district under Tamulpur PHC, four sub-centre areas, viz. Bagrikhuti SC, Hostinapur SC, Citkajan SC and Guwabari SC, with a combined population of 35,526 spread out across 18 villages and 1 tea garden, were included. In the Udalguri district, two PHC areas, viz. Udalguri and Orang, were selected, which included 16 villages and 1 tea garden. The total population under survey was 18,757. All population figures were as per the 2017 data obtained from the district administration.

#### 2.4.3. Deployment in Arunachal Pradesh along the Indo–Myanmar Border

The third phase of the project was initiated in the Changlang district of Arunachal Pradesh along the Indo–Myanmar border. In September 2018, the ICMR-RMRC, N.E. Region, Dibrugarh (Assam) team visited Changlang District Hospital at Changlang, Arunachal Pradesh, and finalised two areas, viz. Changlang District Hospital (DH) and Khimyong PHC. A two-day training program on MoSQuIT software (ver. 1.1) was organised, which was attended by twenty-six ASHAs and two MPWs. Twenty-eight mobile handsets were distributed to the health workers for surveillance. The deployment areas are shown in Supplementary Table S4. Deployment was done in the Changlang District hospital area and Khimyoung PHC area covering a total population of 18,307. The total period of data collection was October 2018 to May 2019.

## 3. Results

**Figure 6:**
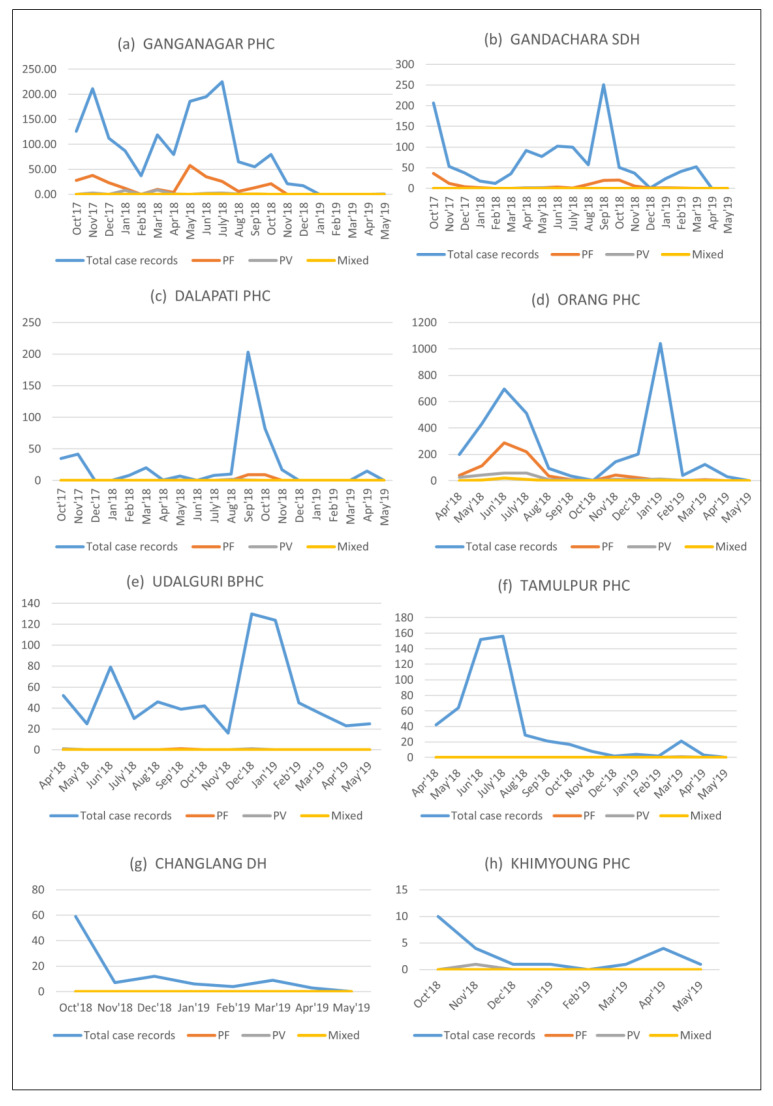
(**a**–**h**) Month-wise distribution of cases across 8 areas in 3 states of Tripura (**a**–**c**), Assam (**d**–**f**) and Arunachal Pradesh (**g**,**h**).

A total of 8221 fever case records were captured by the MoSQuIT app during the deployment period of twenty months. Of these, 1192 cases were *P. falciparum* malaria, 280 *P. vivax* malaria and 52 mixed infections. These data were available in real-time for interpretation and taking appropriate action. The month-wise distribution of the case records in each of the deployment areas is shown in Figure 6a–h below.

### 3.1. Case Records from the Dhalai District of Tripura along the Indo–Bangladesh Border

From Tripura, 3312 fever case records were obtained out of which 411 cases of *P. falciparum* malaria, 37 cases of *P. vivax* malaria and three mixed cases were recorded. From the Ganganagar PHC area, a total number of 1617 case records were obtained during the period October 2017 to May 2019, out of which, 274 *P. falciparum*, 28 *P. vivax* and 3 mixed cases were recorded. In the Gandachara Sub-Divisional Hospital area, 1247 case records were obtained during the same period, out of which, 119 *P. falciparum* and 5 *P. vivax* cases were recorded. A total number of 448 case records were obtained from the Dalapati PHC area during this period, out of which, 18 *P. falciparum* and 4 *P. vivax* cases were reported.

### 3.2. Case Records from Baksa and Udalguri Districts of Assam along the Indo–Bhutan Border

For Assam, the deployment period was 14 months, during which, 4747 fever case records were obtained comprising 781 *P. falciparum*, 242 *P. vivax* and 49 mixed infection cases. In the Baksa district of Assam, villages along the Indo–Bhutan border in the Tampulpur PHC area recorded 521 fever cases, out of which, there was only one confirmed *P. vivax* case. From the Orang PHC area, a total number of 3556 case records were obtained, of which, 780 malaria cases were caused by *P. falciparum* and 239 were caused by *P. vivax*. In the Udalguri BPHC area, a total number of 710 case records were obtained during this period, out of which, one *P. falciparum* and two *P. vivax* cases were recorded.

### 3.3. Case Records from Changlang District of Arunachal Pradesh along the Indo–Myanmar Border

**Figure 7:**
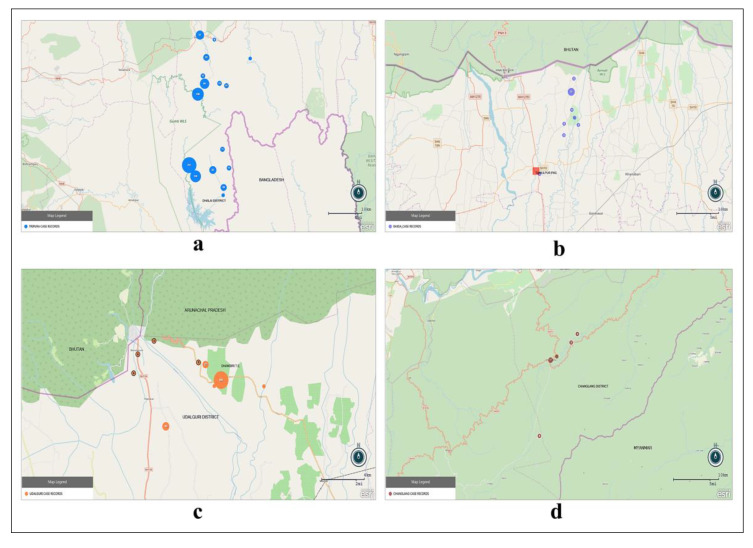
(**a**–**d**) GPS locations of records of fever cases collected in 3 international border areas using MoSQuIT software. (**a**) Dhalai district, Tripura; (**b**) Baksa district, Assam; (**c**) Udalguri district, Assam; and (**d**) Changlang district, Arunachal Pradesh. All maps were designed in EpiInfo ver 7.2.2.6.

In Arunachal Pradesh, during the total deployment period of 8 months, Changlang District Hospital area reported 100 fever cases (no malaria cases recorded) and Khimyoung PHC area reported 22 fever cases, out of which, one *P. vivax* malaria case was documented. GIS-based mapping (in EpiInfo ver 7.2.2.6) of the fever case records in the four districts along three international borders is shown in Figure 7a–d.

## 4. Discussion

### 4.1. Principal Results

Over a combined deployment period of 20 months, the MoSQuIT app was able to collect a total number of 8221 case records. These included *P. falciparum*, *P. vivax* and mixed infections. From this data, a temporal case distribution could be plotted along with the recording of GPS coordinates showing case distribution in the study areas. The mobile-app-based surveillance system resulted in the creation of a continuous and consolidated database in near real time. This was not possible in the conventional paper-based system. It was observed in our on-field surveys that most of the ASHA workers did not submit the full list of cases surveyed with all patient particulars to their respective PHCs on time. With the mobile-based data entry system, this could be done automatically once the data was saved and transmitted. Data collection was structured, with the rapid conversion of data to the digital format occurring in real time. The provision to save data in the offline mode further helped in the collection of case records where the mobile network was not available. The data was transmitted automatically whenever the health worker moved to a location where a mobile network was available. This data could be checked regularly by the district health authorities and verified by the medical officers on the web-based platform. The laboratory reports of slide examination could be conveyed to the ASHA workers through SMS. Data integration was automated at the village, sub-centre, PHC and district levels. Calculation of epidemiological indices was also possible on fortnightly, monthly, quarterly and yearly bases.

### 4.2. Infrastructure Challenges

The MoSQuIT app and web-based system are designed in such a way that the entire database of the case records is available and accessible by all the stakeholders through a user ID and password. During the implementation of the project, ICMR-RMRC, Dibrugarh and C-DAC had provided access to the respective health departments, including the NVBDCP consultants in the four districts. However, some basic infrastructure, such as a desktop computer with a good Internet connection, is mandatory for accessing the database. It was found that Internet access was a problem at Ganganagar PHC and Gandachara SDH in the Dhalai district of Tripura. In Assam, especially in the Tamulpur PHC in Baksa district, infrastructure was a problem too. These basic amenities are necessary if the mobile-based data repository is to be used as a viable alternative to the conventional paper-based system. Moreover, any mobile or web-based system will require a server/cloud for deployment, which involves an additional cost. The smartphones also constitute a major investment in the initial stages; the cost of repairs, battery and screen replacements have to be factored in as well. The mobile-app-based surveillance system provides for geospatial tagging of cases, which can help in the construction of an epidemiological map of case distribution in the study areas. However, there are limitations in the current architecture of the app-based surveillance system. For example, the mobile phones used in the project were mid-range devices that did not have a true in-built GPS. These devices mostly have A-GPS (assisted-GPS), which requires an active mobile data connection for recording the GPS coordinates. In the absence of an active data connection and network availability, the mobile device does not record the GPS coordinates, as is evident from this study, where GPS coordinates were not available for many case records. The mobile network availability in the international border areas in all the three states where the study was implemented was not as good as in other densely populated/plain areas. Even in areas where a mobile network is available, there are intermittent failures, which affect data transmission. The Telecom Regulatory Authority of India (TRAI) reports that at the time of deployment in September 2017, the percentage share of mobile phones in use in Northeast India was only 1.08% (excluding Assam, which was 1.85%) of the total Indian share. Furthermore, this data has not shown any improvement in 2021, with the total share standing at 1.05% for Northeast India. Over the same period, the percentage of wireless connections in Northeast India has also come down by around 3.65%, which is not encouraging [10]. Currently, in Northeast India, Arunachal Pradesh significantly lacks mobile data connectivity, with as many as 64% of the villages in the state having no 3G or 4G mobile Internet [11]. In Northeast India as a whole, it is estimated that as much as 65% of the total population spread out across 8600 villages is still deprived of mobile connectivity [12].

### 4.3. Operational, Scalability and Policy-Level Challenges

The biggest operational challenge in a field-based mobile app-centered surveillance system is that the data collection by the individual health workers is entirely subjective. If the health workers do not enter the data on time or if there is no mobile network, real-time data transmission gets delayed. Literacy rates in NE Indian states are generally above the national average of 74%, except in Assam and Arunachal Pradesh [13]. In our study areas, the educational qualification of the ASHA workers was found to be generally low. They needed constant hand-holding and technical support. Mobile phones are also liable to be stolen and misused. The health workers and other stakeholders also need to be kept motivated for proper adherence to the system. This is especially important because the traditional paper-based system is still being used side-by-side under the programme. Moreover, each state has a different regional language and it would be beneficial if the mobile app can be updated with support for different local languages.

### 4.4. Comparison with Prior Work

The use of digital platforms in the surveillance of vector-borne diseases like malaria is not new [6]. In India, the malaria control system (MCS) used in Mangaluru city, Karnataka, was able to document a significant reduction in malaria incidence in terms of slide positivity rate (SPR) and annual parasite incidence (API) over five years, during which, the system was employed [7]. Similarly, the Solution for Community Health Workers (SOCH) mobile application deployed in the Mandla district of Madhya Pradesh documented a 91% reduction of indigenous malaria cases in the district [8]. These reductions were possible due to regular and timely intervention by the respective health authorities in terms of enforcing vector control measures. However, the establishment of such a temporal association in terms of case numbers was beyond the scope of the current study. Moreover, since the project was implemented in selected border area villages and not entire districts, comparing app data with that of PHC or district-level data was not feasible. In Northeast India, the FeverTracker app, which was deployed in 19 tribal villages in the Dhalai district of Tripura, demonstrated regular data collection over 20 months [9]. The MoSQuIT app used in the current study also documented regular data collection, along with logging of GPS coordinates over the same period. Outside India, in Thailand, the electronic Malaria Information System (eMIS) has shown improved quality as a malaria surveillance system. The system (eMIS) demonstrated good levels of simplicity, stability, acceptability and flexibility [14]. The current study showed that the conventional data collection and surveillance records related to malaria surveillance can be vastly improved by the adoption of a mobile-app-based system in terms of maintaining a centralised and easily accessible data repository that can be utilised for data analysis and the generation of epidemiological reports in near real time. The additional advantage of recording the GPS coordinates enables the creation of geospatial maps for accurate case distribution, which is not available in the current conventional paper-based system. In Cambodia, the existing Malaria Information System (MIS) was upgraded to a web-based platform with new functionalities, such as data visualisation and other unique features specific to the country [15]. Such upgrades and modifications are essential in any application to make way for newer and improved technologies and to ensure wider acceptability. The MoSQuIT app has undergone many changes and the interface has been modified since it was first piloted in 2012 in Dibrugarh district, Assam [5]. The system also has an inbuilt outbreak predictor, which can be initialised by a local health worker when they encounter an increased number of cases from a particular region. This will alert the concerned health officials, who can then initiate a quick response. Similar steps were adopted for reporting disease outbreaks. In Kenya, the mSOS (mobile SMS-based disease outbreak alert system) was used by the Ministry of Health in 2013–2014 for tracking 12 immediately notifiable diseases [16]. Apart from human health, in veterinary sciences, smartphone-based apps have shown increased accuracy and faster data transmission times compared to traditional paper-based surveillance systems [17]. Malaria surveillance data through the traditional paper-based systems usually take 15–30 days to reach the health authorities and the picture is similar in most parts of India [8]. When using mobile-based systems, such as the MoSQuIT app, this time can be reduced drastically. However, in remote hilly areas, such as Northeast India, the transmission of data may be hampered by mobile network limitations. Moreover, the availability of electricity to charge mobile phones regularly can delay data transmission times as well.

### 4.5. Roadmap for the Future

The MoSQuIT app and web-based system can be upscaled for deployment in entire districts and states in the future. The system architecture can be customised to include other vector-borne diseases and for surveillance of both communicable and non-communicable diseases with public health importance. The system can also be integrated into machine-learning-based predictive models. Retrospective data collection and data collection from the private sector can also be integrated with the system to obtain a robust surveillance tool.

## 5. Conclusions

The present study demonstrated that malaria surveillance data can be transmitted to the stakeholders in a matter of minutes from remote areas compared with days under the conventional paper-based surveillance system. Additionally, the centralised data repository created is available for viewing and analysis using role-based access control. Geospatial logging of case records helps with the easy visualisation of case distribution for the identification of highly malaria-prone areas. However, to achieve the full potential of the mobile-app-based system, a good Internet connection is a must. This is challenging in remote international border areas. Additionally, the entire data collection and transmission process is dependent on the diligence of the health workers. If the health workers do not enter the records promptly and/or a good mobile network is not available, then real-time surveillance is hampered. Keeping these points in mind, the mobile-based malaria surveillance system can be scaled up further to include more vector-borne diseases in wider geographical areas with coverage of entire districts/states in a phase-wise manner. It can be fully integrated with the existing surveillance system under NVBDCP with the participation of the state and central health authorities and ministries. Mobile network coverage is also improving daily and health workers are now more conversant with the use of smartphones. Wide-scale implementation and increased acceptance at the health worker level will require additional modifications in the existing architecture of the mobile application in terms of incorporating local languages and more user-friendly pictorial data entry systems, both of which can be achieved in the future.

## Data Availability

All data reported in this study are available in the local server at ICMR-RMRC North East Region, Dibrugarh.

## Supplementary Materials

The following supporting information can be downloaded at: https://www.mdpi.com/article/10.3390/ijerph19052561/s1, Table S1: Deployment areas in Tripura along the Indo–Bangladesh border; Table S2: Deployment areas in Baksa district, Assam; Table S3: Deployment areas in Udalguri district, Assam; Table S4: Deployment areas in Changlang, Arunachal Pradesh.
