# Supplementary material for: Development and On-Field Deployment of a Mobile-Based Application ‘MoSQuIT’ for Malaria Surveillance in International Border Districts of Northeast India—Challenges and Opportunities
Source: Int J Environ Res Public Health. 2022 Feb 23;19(5):2561. doi: 10.3390/ijerph19052561 (PMC8909168; doi:10.3390/ijerph19052561)
Supplement: Supplementary file 1 [file ijerph-19-02561-s001.zip › ijerph-1577138-Supplementary.pdf]

Table S1: Deployment areas in Tripura along the Indo-Bangladesh border.

Ganganagar PHC		
**Name of the Sub-center**	**Para/Village Name**	**Population**
Malda Kumar Para	Dantipara, M. K. Para 1, M. K. Para 2, Anandakumar RP, Bhubanda Kumar RP, Ruhida para, Jatindra para.	2665
Karnamoni Para	Karnamoni Para, Champarai, Budhajoy para, Joyrampara, Baluchara, Potiraipara, 10 mile para, karanjoy para, Purnajoypara, Parbomonipara, Nunachara.	3049
Gandachara SDH		
**Name of the Sub-center**	**Para/Village Name**	**Population**
Bhagirathpara	Girachandrapara, Nabakumarpara, Dhanurampara, Natun Bhagirath Colony, Birkumarpara, Old Bhagirathpara.	1237
Wanasapara	Rajdhanpara, Premkumarpara, New joyrampara, Old Joyrampara, Wanasapara, Kuichangrai, Duisharampara.	2944
Ratan nagar	Majhimonipara, Aswanikumarpara, Anandamohan RP, SharadaRoajapara,Dhamonipara, Sen kumar para, Mohinikumar para	3539
Dalapati PHC		
**Name of the Sub-center**	**Para/Village Name**	**Population**
Dalapati	Madhuhari para, Old Dalapati para, New Dalapati para, Sira para, Nabdip para, Biswamohan para, Karan Kishor para, Gahurchau para, Jam para, Kishan Ch para	3000

Table S2: Deployment areas in Baksa district, Assam.

**PHC**	**Bagrikhuti SC**	**Total Population/2017**	**Villages**	**Population**
Tamulpur PHC	API=2.39	7132	Orangajuli	1321
			No 1 Bagrikhuti	1878
			No 2 Bagrikhuti	2443
			Joypur	1490
PHC	Hostinapur SC	Total Population	Villages	Population
Tamulpur PHC	API=0.18	11093	Hostinapur	1664
			Mohendranagar	1571
			2 No Dangargaon	1349
			Nagrijuli T.E.	3012
			Dakshin Dangargaon	1153
			Kalcheni	1160
			Piplani	1184
PHC	Citkajan SC	Total Population	Villages	Population
Tamulpur PHC	API=0.00	5242	Citkajan	1702
			Bimalanagar	1222
			Natunkhuti	1114
			Ekra bil	1204
PHC	Guwabari SC	Total Population	Villages	Population
Tamulpur PHC	API=0.00	4339	Pub Guwabari	1205
			Boro Basti	954
			Pachim Guwabari	1121
			Hatikata	1059

Table S3: Deployment areas in Udalguri district, Assam

PHC	Dalanabasti S/C	Total Population/2017	Villages	Population
Orang PHC	API>5	8556	Paharpur, Sikaridanga	1050
			Dalanibasti	1279
			Lalpani 3	293
			NK Dhansiri	1112
			Monai/Thakurpar	
			Bagaribari/Onthaibari	1500
			Dhansiri TE	3322
PHC	Bhariabkunda S/C		Villages	Population
Udalguri PHC	API=1	6023	Bhairabpur	1482
			Angrajuli	1376
			Jamuguri	750
			Mazargaon	1073
			Bhoirabkunda	1342
PHC	Pakribari S/C		Villages	Population
Udalguri PHC	API=1.52	4178	Pakribari	983
			Gerua	551
			Daoduigaon	639
			No 3 Amjuli	1011
			Amsigiri	994

Table S4: Deployment areas in Changlang, Arunachal Pradesh.

**PHC**	**DH Changlang**	**Total Population**	**Villages**	**Population**
DH Changlang	API=2.70	11462	Changlang Town	6945
			Others	4517
PHC	Kengkhu SC	Total Population	Villages	Population
Changlang D/H	API=0.40	2391	Simrang	216
			Kengkhu	410
			Rangkatu	313
			Kengkhu Tea Estate	350
			New Rangkatu	121
			Chokdok –I	63
			Chokdok – II	214
			Jungmaisung	153
			Kuchep-I	333
			Kuchep-II	218
PHC	Phangtip SC	Total Population	Villages	Population
Changlang D/H	API=8.11	988	Wawoi Sabban	197
			Phangtip	205
			Jongpho Hate	359
			Simnom	36
			Hatangsu	94
			Satkhetong	97
PHC	Kangtang SC	Total Population	Villages	Population
Changlang D/H	API=7.59	791	Hadap	124
			Chimsu	94
			New Lunglung	141
			Sikeng	26
			Kantang	112
			College Campus	92
			Lunglung	202
PHC	Khimyoung PHC	Total Population	Villages	Population
Khimyoung	API=2.61	1533	Khimyoung Headquarter	137
			Khimyong village	381
			Longkey	306
			Thamlom	235
			Wafang	63
			Phungsa	163
			New Lonkey	43
			Changrah	205
PHC	Yanman SC	Total Population	Villages	Population
Khimyoung	API=3.44	1142	Yanman	585
			Yanchang	100

			Hanalthung	188
			Thamiyang	184
			Datkan	85
